# Clonal hematopoiesis is associated with adverse events in patients with high-grade carotid artery stenosis

**DOI:** 10.1016/j.athplu.2026.100617

**Published:** 2026-10-01

**Authors:** Olesya Harkot, Roland Jäger, Stephanie Kampf, Michael Gurbisz, Maja Carina Nackenhorst, Johann Wojta, Christian Hengstenberg, Bernhard Haring, Harald Esterbauer, Christoph Neumayer, Stefan Stojkovic, Svitlana Demyanets

**Affiliations:** a Division of Cardiology, Department of Internal Medicine II, Medical University of Vienna, Vienna, Austria; b Department of Laboratory Medicine, Medical University of Vienna, Vienna, Austria; c Division of Vascular Surgery, Department of Surgery, Medical University of Vienna, Vienna, Austria; d Department of Pathology, Medical University of Vienna, Vienna, Austria; e Ludwig Boltzmann Institute for Cardiovascular Research, Vienna, Austria; f Core Facilities, Medical University of Vienna, Vienna, Austria; g Department of Medicine IV, Clinic Hietzing, Vienna Healthcare Group, Vienna, Austria; h Department of Laboratory Medicine, Clinic Hietzing, Vienna Healthcare Group, Vienna, Austria

**Keywords:** Atherosclerosis, Cardiovascular disease, Hematopoiesis, Somatic mutations, Clonal expansion

## Abstract

**Introduction:**

Clonal hematopoiesis (CH), defined as the expansion of a subset of hematopoietic stem or progenitor cells driven by acquired mutations, represents a novel cardiovascular risk factor. Its role in patients with high-grade carotid stenosis remains to be evaluated.

**Patients and methods:**

Next-generation sequencing (NGS) involving 30 genes implicated in CH was used as diagnostic-grade assay (SOPHiA DDM Myeloid Solution) to screen for CH in 83 prospectively collected patients with symptomatic or asymptomatic high-grade carotid stenosis undergoing carotid endarterectomy (CEA).

**Results:**

Clonal markers were detected in 16 (19.7 %) of 83 patients (median age 71.2 years), with prevalence increasing at advanced age. At 5-year follow-up, CH carriers showed a higher cumulative incidence of major adverse cardiovascular events (MACE; P = 0.040) compared to patients without CH; all-cause mortality did not differ significantly between groups (P = 0.57). Cox regression revealed a significant association between CH and MACE in a univariable model (P = 0.047), which remained significant after multivariable adjustment for age and sex (P = 0.018). Exploratory analyses suggested that the association between CH and MACE may differ according to carotid stenosis severity, with an increased incidence of MACE observed in patients with <90 % stenosis and concomitant CH, an effect absent in patients with ≥90 % stenosis (*P*_interaction_ = 0.03).

**Discussion and conclusions:**

CH was associated with an adverse 5-year outcome in patients with carotid artery stenosis. These findings identify CH as a hematologic contributor to post-interventional cardiovascular risk and indicate that assessment of CH may offer additional prognostic information for residual risk stratification.

## Highlights

• CH was present in ∼20 % of patients undergoing carotid endarterectomy.
• CH predicted major adverse cardiovascular events after adjustment for age and sex.
• Risk associations were most pronounced in patients with <90 % carotid stenosis.
• CH assessment may provide additional prognostic value for residual cardiovascular risk stratification.

## Graphical abstract

**Figure:**
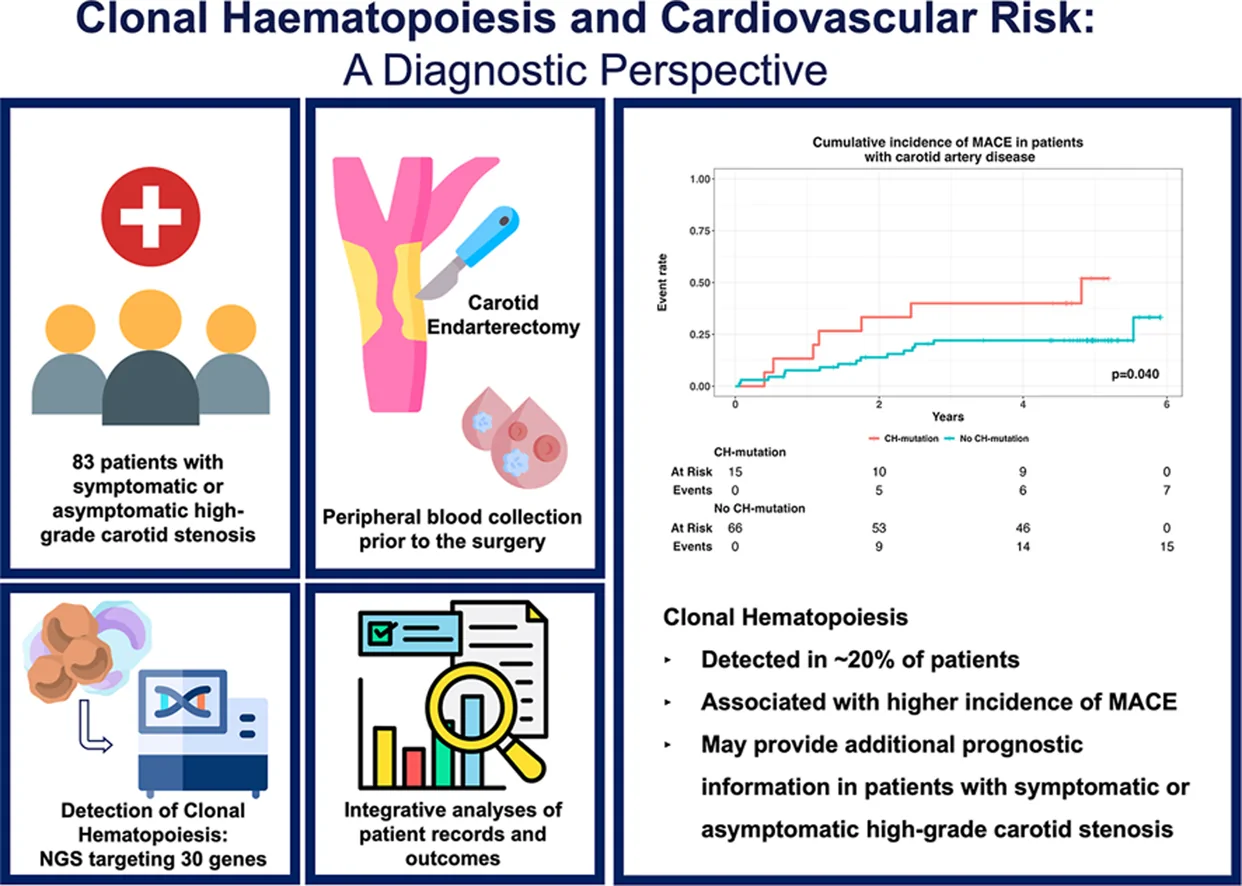

## 1 Introduction

Carotid atherosclerosis is responsible for 15-20 % of all strokes of ischemic origin, therefore representing a strong risk factor for one of the leading causes of death around the world [1]. Tools for risk stratification of patients with high-grade carotid stenosis are limited and include circulating biomarkers and imaging modalities such as molecular imaging [[2], [3], [4], [5], [6]]. Effective risk stratification is essential to guide both primary and secondary prevention as well as postoperative management in patients following carotid endarterectomy (CEA). Unfortunately, current approaches remain limited, as they rely largely on anatomical and clinical parameters that do not fully capture the biological heterogeneity or molecular determinants of recurrent plaque instability and cardiovascular risk.

Clonal hematopoiesis (CH) is generally defined as the clonal expansion of a subset of hematopoietic stem or progenitor cells due to acquired mutations, whereas CH of indeterminate potential (CHIP) additionally implies the absence of features defining hematological malignancies [7,8]. Presence of CH/CHIP has been associated with increased cardiovascular events and mortality in the general population [9], as well as in patients with ASCVD such as CAD, ischemic stroke, and/or peripheral artery disease (PAD) [10] and patients with heart failure (HF) [11]. While subgroup analyses revealed associations with increased risk of stroke to be particularly pronounced in hemorrhagic and small vessel ischemic stroke [12], Mayerhofer et al. recently reported on an association of CHIP with carotid intima media thickness and aortic plaques in younger stroke patients [13]. Of note, functional studies in mouse models provide robust evidence for a crucial pathogenic role of proinflammatory clonal leukocytes in mediating the clinical impact of CHIP [9,14,15]. In line with that, monocyte-derived macrophages carrying CHIP-associated mutations were visualized in atherosclerotic plaques in carotid artery lesions [16] and human coronary arteries [17]. Moreover, carotid plaque composition has been recognized as a determinant for the secondary atherosclerotic disease manifestation and progression [18]. Whereas a potentially bidirectional interplay of hyperinflammation and clonal expansion is matter of discussion [19], most recently a unidirectional association of CH with atherosclerosis development was reported in a longitudinal cohort of healthy middle-aged subjects [20].

While CEA effectively reduces the risk of ipsilateral stroke, patients remain at substantial residual risk for major adverse cardiovascular events (MACE) [21], reflecting the systemic nature of atherosclerosis. Currently, there are limited tools to identify patients at increased cardiovascular risk after successful plaque removal. Using a real-world post-CEA cohort, we hypothesized that CH, as detected by a 30-gene diagnostic NGS assay, is prevalent in patients with high-grade carotid artery stenosis and is independently associated with residual cardiovascular risk.

## 2 Patients and methods

### 2.1 Patient cohort

In this study, 83 consecutive patients with carotid artery stenosis who underwent CEA were enrolled. All surgical procedures were conducted at the Department of Vascular Surgery, Medical University of Vienna, Austria. The study received approval from the Ethics Committee of the Medical University of Vienna (votum #2449/2020) conforming to the principles indicated in the Declaration of Helsinki, and all participants provided written informed consent.

Indications for surgery included symptomatic carotid artery stenosis or high-grade asymptomatic stenosis (≥70 %). The degree of luminal narrowing was assessed using carotid duplex sonography and/or computed tomography angiography, following the criteria of the North American Symptomatic Carotid Endarterectomy Trial [22]. In our cohort, asymptomatic patients with carotid stenosis had a mean luminal narrowing of 85 % (±12 %). Symptomatic patients showed a mean luminal narrowing of 82 % (±17 %). Symptomatic patients were defined as those who had experienced a stroke, transient ischemic attack, or amaurosis fugax ipsilateral to the carotid artery stenosis within six months prior to the procedure. Exclusion criteria included hemorrhagic, lacunar, or cardioembolic strokes. Lacunar strokes were characterized by primary motor, primary sensory, or sensory-motor symptoms along with deep white-matter or basal ganglia lesions measuring 1 cm or less. Suspected cardioembolic strokes in patients with atrial fibrillation, recent myocardial infarction, unstable angina, recent congestive heart failure, and valvular disease were reviewed by an independent cardiologist. None of the individuals showed signs of haematological malignancy or had a history of haematological malignancies.

Patients were followed-up for a median of 4.8 (range 2.4-5.1) years post-surgery. In this study, the primary cardiovascular endpoint is represented by occurrence of MACE. MACE was defined as a composite of cardiovascular death, acute myocardial infarction (AMI), transient ischemic attacks (TIAs), or stroke, as well as progression of atherosclerosis in the coronary or peripheral arteries necessitating either interventional (percutaneous coronary intervention or peripheral balloon angioplasty with or without stenting) or surgical revascularization (aortocoronary bypass or peripheral bypass). All-cause mortality was designated as a secondary endpoint. Survival status was ascertained through linkage with the institutional medical records system.

Plaques were classified based on ultrasound echogenicity into echolucent (non-calcified), echogenic (calcified), and mixed (partially calcified) categories at the expert vascular surgery laboratory of the Medical University of Vienna. For histological classification, endarterectomy specimens were fixed in formalin and embedded in paraffin. Experienced technicians cut transverse sections at 3 mm intervals along the plaque. These sections were stained with hematoxylin-eosin and elastic-van-Giessen staining and classified according to a modified American Heart Association (AHA) classification based on morphological characteristics [23]. Whole peripheral blood (PB) samples required for study-specific analyses were collected one day prior to surgery.

### 2.2 High-throughput sequencing and variant detection

A commercial, diagnostic-grade gene panel (Myeloid Solution; SOPHIA Genetics, Lausanne, Switzerland) was used to sequence relevant regions in 30 genes frequently mutated in myeloid neoplasia and in CH, including main CH drivers. The NGS assay involved all exons or selected exons of the genes *ABL1, ASXL1, BRAF, CALR, CBL, CEBPA, CSF3R, DNMT3A), ETV6, EZH2, FLT3, HRAS, IDH1, IDH2, JAK2, KIT, KRAS, MPL, NPM1, NRAS, PTPN11, RUNX1, SETBP1, SF3B1, SRSF2, TET2, TP53, U2AF1, WT1*, and *ZRSR2*. DNA was extracted from patients' PB using the QIAsymphony DSP DNA Kit (Qiagen, Hilden, Germany). Subsequent to NGS library preparation accomplished according to the manufacturer's instructions, samples were sequenced on MiSeq instrument (Illumina, San Diego, USA) generating 300 base-pair paired-end reads to cover target regions to a minimum of 1000x. Massive parallel sequences were aligned and processed using the SOPHIA DDM software (SOPHIA Genetics, Lausanne, Switzerland). Annotated variants were filtered according to the following inclusion criteria: variant site depth (≥1000), variant reported as somatic in COSMIC [24], population allele frequency <0.1 % in gnomAD databases [25], non-synonymous substitution or insertion/deletion in protein-coding sequence. Candidate somatic variants were additionally restricted to variant allele frequencies (VAFs) between 2 % and 40 % or between 60 % and 90 %. Variants exhibiting allele frequencies outside of those ranges were excluded from analysis to avoid inclusion of private germline variants in the absence of non-blood germline control samples.

### 2.3 Statistics

Categorical variables were summarized as counts and percentages and compared using the χ^2^-test or Fisher's exact test, as appropriate. Continuous variables were reported as medians with interquartile ranges (IQR) and compared using the *t*-test or one-way ANOVA. For non-normally distributed data, the Mann-Whitney *U* test was applied. Associations of CH with baseline clinical presentation (symptomatic vs. asymptomatic) and carotid plaque morphology (ultrasound echogenicity and histological AHA classification) were assessed using Fisher's exact test or chi-squared test, as appropriate. Additionally, a univariable and multivariable Cox proportional hazard regression models were used to evaluate whether CH could predict long-term cardiovascular outcomes. To assess effect modification by stenosis severity, we fitted Cox models including an interaction term between CH and stenosis grade at a 90 % cutoff. Interaction was evaluated using Wald and likelihood-ratio tests (LRT). Events-per-variable (EPV) ratios were calculated for each multivariable Cox model as the number of observed MACE events divided by the number of covariates included. All models used complete-case analysis, the analytic sample therefore varies by model according to covariate availability (Supplemental Table 1). Kaplan-Meier analysis was conducted to compare the time-dependent discriminative power of CH. Statistical significance was determined with two-sided p-values of 0.05. All statistical analyses and visualizations were conducted using SPSS version 22.0 (IBM Corporation, Armonk, NY, USA), R programming environment version 4.02, and Python programming environment version 3.8.6.

## 3 Results

### 3.1 Patient characteristics and detection of clonal hematopoiesis

A total of 83 patients referred to CEA with high-grade stenosis underwent targeted re-sequencing involving 30 genes to determine the presence of CH. Median age of the study participants was 71.2 years (interquartile range (IQR) 67.4-76.7 years) at the time of inclusion, and 32 (39 %) patients were female. Detailed demographic and clinical characteristics of included patients are provided in Table 1.

**Table 1:** Clinical characteristics of the patient cohort.

	Overall (n = 83)	No CH (n = 67)	CH (n = 16)	P-value
**Demographics**				
Age	71 (67-77)	71 (66-77)	72 (68-76)	0.514
Sex (female)	32 (39 %)	24 (36 %)	8 (50 %)	0.447
**Characteristics of carotid artery stenosis**				
Grade of stenosis	90 (80-95)	90 (78-95)	85 (80-95)	0.974
Stenosis grade ≥90 %	40 (48 %)	34 (51 %)	6 (38 %)	0.493
Contralateral stenosis	18 (22 %)	13 (19 %)	5 (31 %)	0.437
**Plaque morphology**				
Echogenic	28 (34 %)	23 (34 %)	5 (31 %)	0.148
Mixed	4 (5 %)	2 (3 %)	2 (12 %)	
Echolucent	34 (41 %)	30 (45 %)	4 (25 %)	
**Histological AHA classification**				
Type V fibroatheroma	12 (14 %)	10 (15 %)	2 (12 %)	0.893
Type VI complex lesion	13 (16 %)	11 (16 %)	2 (12 %)	
Type VII calcified lesion	12 (14 %)	10 (15 %)	2 (12 %)	
Type VIII fibrotic lesion	7 (8 %)	5 (7 %)	2 (12 %)	
**Outcome after CEA**				
Restenosis	7 (8 %)	4 (6 %)	3 (19 %)	0.131
TIA/STROKE	5 (6 %)	3 (4 %)	2 (12 %)	0.250
CAD progression	6 (7 %)	4 (6 %)	2 (12 %)	0.326
**Comorbidities and risk factors**				
Transient ischemic attack	12 (14 %)	8 (12 %)	4 (25 %)	0.233
Stroke	10 (12 %)	10 (15 %)	0 (0 %)	0.197
History of stroke/TIA	17 (20 %)	14 (21 %)	3 (19 %)	1.000
Acute myocardial infarction	8 (10 %)	8 (12 %)	0 (0 %)	0.340
CAD	25 (30 %)	21 (31 %)	4 (25 %)	0.765
PAD	26 (31 %)	19 (28 %)	7 (44 %)	0.393
Arterial hypertension	76 (92 %)	62 (93 %)	14 (88 %)	1.000
Hyperlipidaemia	71 (86 %)	59 (88 %)	12 (75 %)	0.383
DM type 2	25 (30 %)	20 (30 %)	5 (31 %)	1.000
Obesity (BMI>30)	21 (25 %)	14 (21 %)	7 (44 %)	0.117
Active smoking status	22 (27 %)	19 (28 %)	3 (19 %)	0.502
Past smoker	39 (47 %)	31 (46 %)	8 (50 %)	1.000
Pack-years	18 (0-40)	20 (0-40)	10 (0-21)	0.466
Previous cancer	9 (11 %)	5 (7 %)	4 (25 %)	0.068
**Medications**				
ACE inhibitors	33 (40 %)	27 (40 %)	6 (38 %)	1.000
Angiotensin receptor blockers	39 (47 %)	31 (46 %)	8 (50 %)	0.874
Beta-blockers	38 (46 %)	30 (45 %)	8 (50 %)	0.791
Acetylsalicylic acid	63 (76 %)	49 (73 %)	14 (88 %)	0.172
Clopidogrel	28 (34 %)	26 (39 %)	2 (12 %)	0.075
NOAC	8 (10 %)	6 (9 %)	2 (12 %)	0.637
Metformin	14 (17 %)	12 (15 %)	2 (2 %)	1.000
Statins	79 (95 %)	64 (96 %)	15 (94 %)	1.000
**Laboratory parameters**				
Total cholesterol, mg/dL	152 (125-179)	156 (131-179)	148 (122-181)	0.971
LDL, mg/dL	67 (53-95)	67 (53-94)	64 (54-98)	0.772
HDL, mg/dL	49 (38-60)	49 (38-63)	50 (40-57)	0.561
Triglycerides, mg/dL	131 (102-194)	132 (102-197)	124 (98-150)	0.325
High-sensitivity CRP, mg/dL	0.25 (0.11-0.53)	0.23 (0.12-0.64)	0.32 (0.1-0.4)	0.726

Continuous data are shown as median (interquartile range). Dichotomous data are shown as n (%). Mann-Whitney test was used for the statistical comparison of continuous variables between symptomatic and asymptomatic plaques and Fisher's exact test for the categorical variables. The Chi-square test was used for categorical variables of more than two classes (ultrasound and histology). All patients of the cohort were Caucasians. Abbreviations: ACE: Angiotensin-Converting-Enzyme; AHA, American Heart Association; CAD: coronary artery disease; CEA, carotid endarterectomy; BMI, body mass index; CRP, C-reactive protein; DM: diabetes mellitus; HDL, high-density lipoprotein; LDL, low-density lipoprotein; NOAC, new oral anticoagulants; PAD: peripheral artery disease; TIA, transient ischemic attack. No corrections for multiple testing were applied.

We detected somatic mutations in 16 (19.7 %) out of 83 patients. In total, 20 mutations were identified, occurring across 9 of the 30 genes analyzed. The vast majority (87.5 %) of patients with CH carried only one mutation, whereas a single patient carried two mutations, and another patient carried three mutations (Supplemental Figure 1A). Consistent with previous studies, the prevalence of CH in our cohort increased with patient age (Fig. 1A). Clonal markers were detected in 9.1 % of patients under 60 years of age (1 of 11 patients), 22.7 % of patients between age 60 and 69 years (5 of 22 patients), and 17.5 % between age 70 and 79 years (7 of 40 patients). In patients older than 80 years (n = 10), CH was detected in three individuals, corresponding to a CH rate of 30.0 % (Fig. 1A). A test for linearity across categories indicated a non-significant trend ( *P*= 0.4), likely reflecting the limited sample size.

**Fig. 1:**
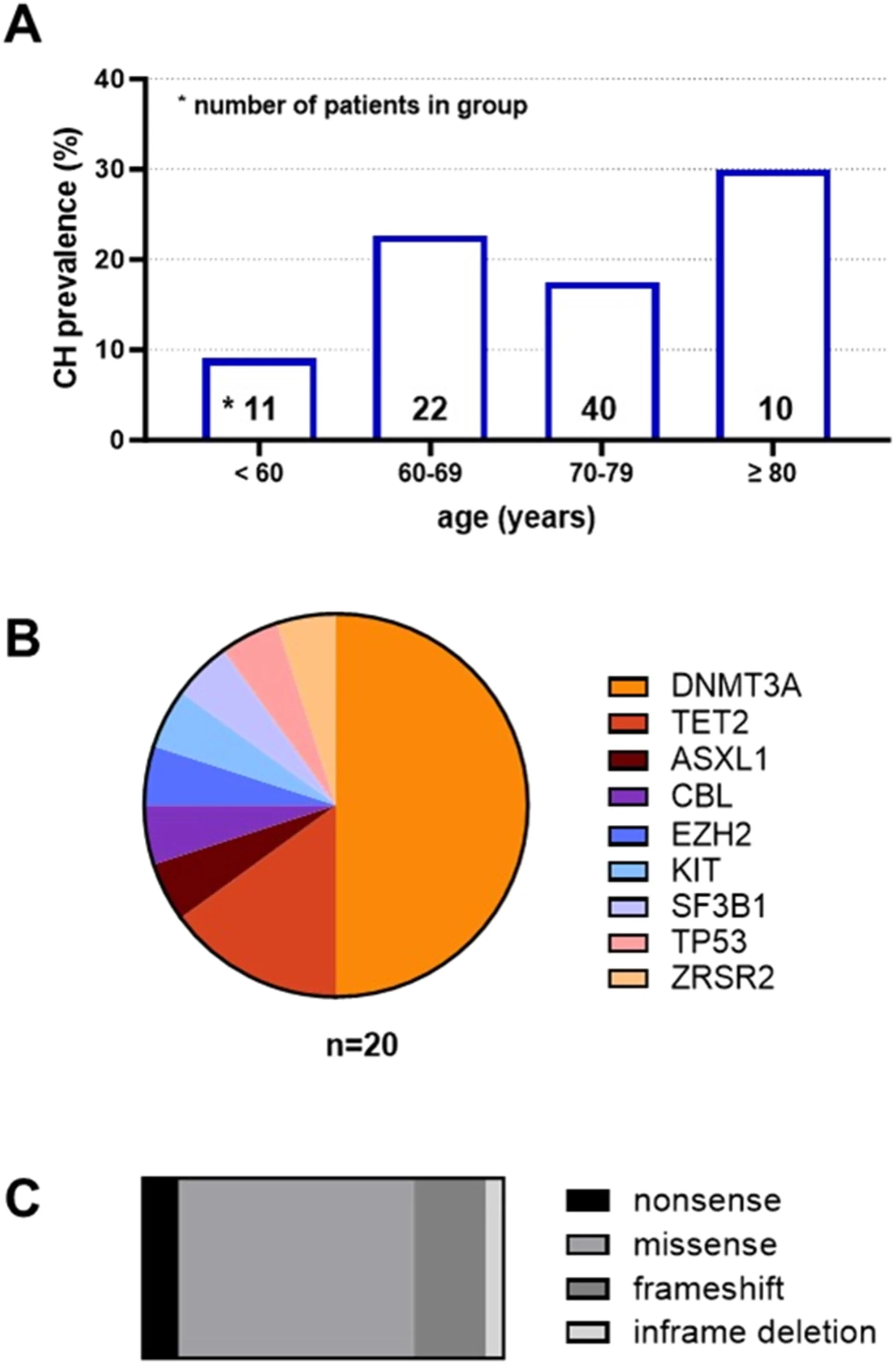
**Somatic mutations underlying clonal hematopoiesis in patients with high-grade carotid artery stenosis. (A)** Prevalence of clonal hematopoiesis (CH) according to age. Blue bars show the frequency of CH in age groups binned as outlined on the x-axes. Numbers in bars represent the total count of individuals in age groups, totaling to 83 patients investigated. **(B)** Frequency distribution of mutations (n = 20) in individual genes (n = 9). **(C)** Type of somatic variants identified overall, grouped into nonsense (premature stop codon), missense (single amino-acid exchange) and frameshift mutations as well as inframe deletions.

The vast majority of mutations were detected in *DNMT3A* (50.0 %) and *TET2* (15.0 %). Furthermore, one mutation each was observed in the genes *ASXL1, CBL, EZH2, KIT, SF3B1, TP53*, and *ZRSR2* (each 5 % of total mutations; Fig. 1B). Most variants detected represent missense mutations in line with previous observations in cohorts with *DNMT3A* as predominant driver of CH [26], followed by nonsense and frameshift variants likely resulting in loss-of-function typically observed in tumor suppressor genes such as *TET2* (Fig. 1C). The majority of mutations in all 30 investigated genes had low VAFs between 2 % and 10 %, corresponding to clonal fractions of approximately 4 % to 20 % for heterozygous mutations (Supplemental Figure 1B).

### 3.2 Association of clonal hematopoiesis with adverse events

Following the surgical intervention, MACE was observed in 27 % of patients for the median follow-up of 4.8 (IQR 2.4-5.1) years. Kaplan-Meier analysis did not reveal a statistically significant difference for all-cause mortality (log-rank *P* = 0.57). Cox proportional hazard regression analysis was fit to assess the predictive value of CH and other clinical parameters for MACE, revealing a statistically significant association between CH and MACE in a univariable model (Table 2). Other relevant clinical parameters significantly associated with MACE in our cohort were female sex, active smoking status, diabetes mellitus (DM), and PAD, whereas age showed borderline significance (Table 2). In Kaplan-Meier analyses, CH carriers exhibited a significantly higher cumulative incidence rate of MACE as primary endpoint compared to patients without CH (log-rank *P* = 0.040; Fig. 2.

**Table 2:** Cox Proportional Hazard Regression Univariable Analysis to Assess the Association Between MACE and relevant clinical parameters.

Covariate	HR per 1-SD	95 % CI	P value
Age	0.69	0.46−1.02	0.064
Sex	1.62	1.07−2.44	**0.023**
Symptomatic Carotid Stenosis	0.88	0.56−1.38	0.581
Obesity	0.95	0.61−1.48	0.832
AMI	1.20	0.81−1.77	0.363
CAD	0.78	0.48−1.29	0.334
PAD	1.63	1.09−2.42	**0.016**
Hypertension	1.01	0.69−1.48	0.978
Hyperlipidaemia	1.49	0.76−2.90	0.246
DM	1.74	1.16−2.58	**0.007**
Active Smoking Status	1.78	1.07−2.95	**0.026**
COPD	0.89	0.53−1.51	0.665
Total Cholesterol	0.88	0.55−1.40	0.581
LDL	0.78	0.45−1.34	0.365
HDL	0.82	0.51−1.30	0.395
Triglycerides	1.35	0.93−1.97	0.112
hsCRP	0.95	0.59−1.53	0.840
Clonal Hematopoiesis	1.43	1.00−2.04	**0.047**

Abbreviations: AMI, acute myocardial infarction; CAD: coronary artery disease; COPD: chronic obstructive pulmonary disease; DM: diabetes mellitus; HDL, high-density lipoprotein; hsCRP, high-sensitive C-reactive protein; LDL, low-density lipoprotein; PAD: peripheral artery disease. No corrections for multiple testing were applied.

**Fig. 2:**
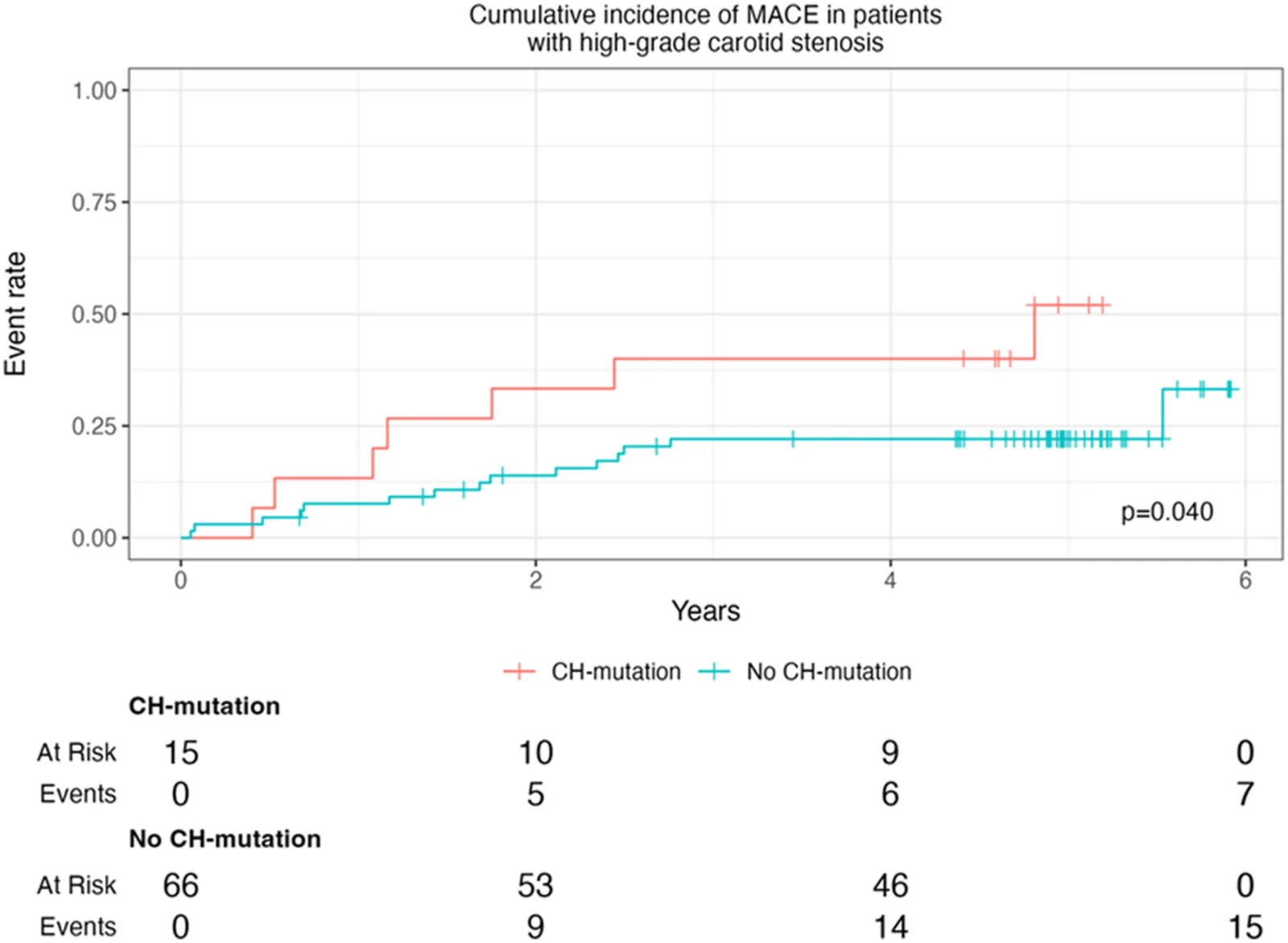
**The Effect of CH on MACE.** Cumulative incidence of MACE according to presence (red line) or absence (blue line) of CH during median follow up time of 4.81 years.

While in univariable Cox regression CH predicted MACE with Hazard Ratio (HR) 1.43 (95 % Confidence Interval (CI) 1.00-2.04; *P* = 0.047; Fig. 3 and Supplemental Figure 2), multivariable analyses including age and sex as covariates (Model 1) revealed CH as independent predictor of MACE with HR 1.57 (95 % CI 1.08 – 2.28; *P* = 0.018; Fig. 3 and Supplemental Figure 3). In a full multivariable model including all covariates that were statistically significant in the univariable analysis and age (Model 2), the association was no longer statistically significant (Fig. 3 and Supplemental Figure 4). Given the low EPV (2.5), this model should be interpreted with caution because it is likely underpowered and at substantial risk of overfitting.

**Fig. 3:**
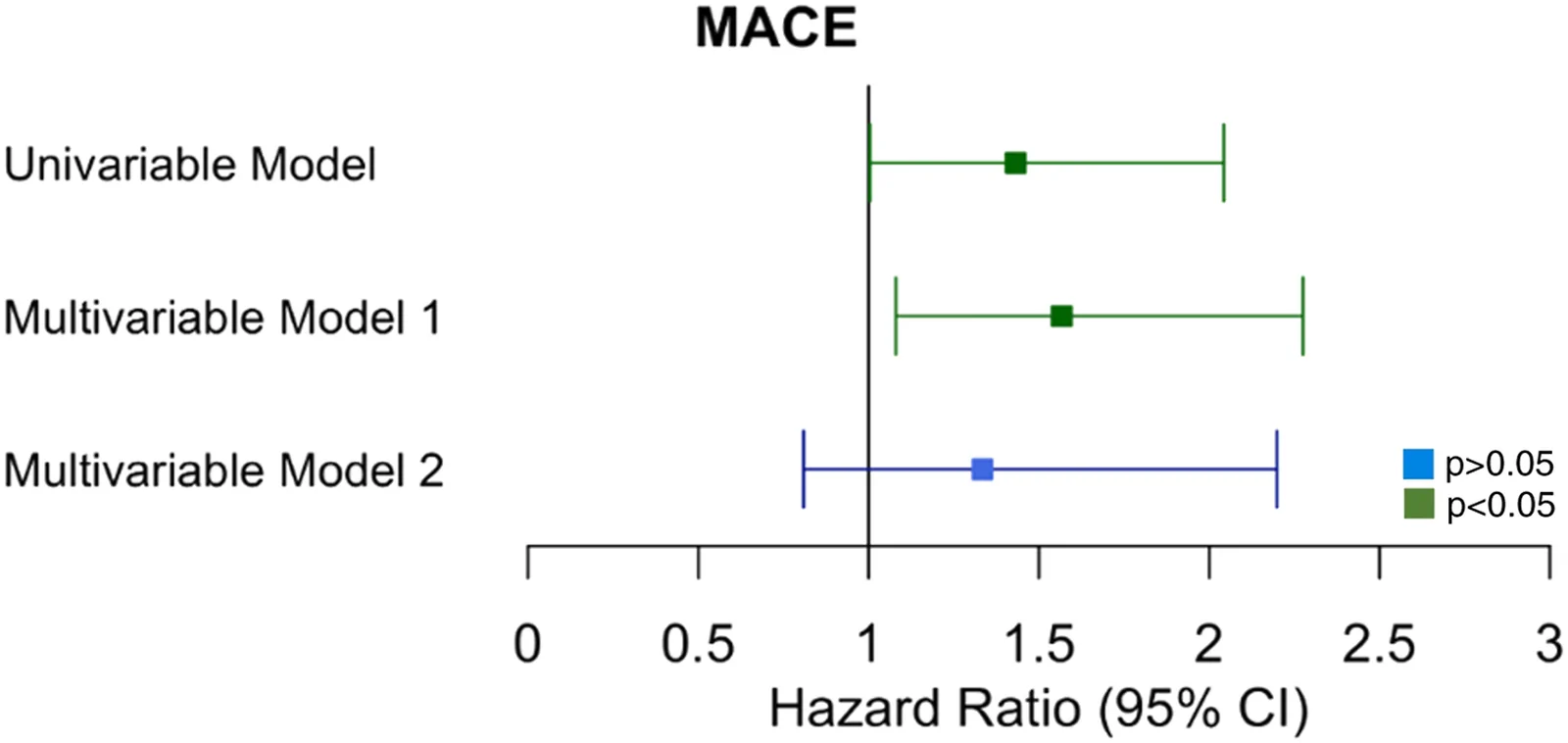
**Cox proportional hazard regression analyses to assess the association between MACE and CH.** Multivariable model 1 adjusted for age and sex. Multivariable model 2 adjusted for age, sex, peripheral artery disease, diabetes mellitus and active smoking status. Shown are hazard ratios (HRs) per 1-SD for MACE in conjunction with 95 % confidence intervals (CI).

To reduce the risk of overfitting, we employed additional multivariable models (Models 3-5; Supplemental Table 1), each including age and sex together with one additional relevant covariate (smoking, PAD, or DM), resulting in a total of three covariates per model. This approach allowed us to evaluate the individual contribution of each covariate to the association between CH and outcome while minimizing model complexity. In the smoking-adjusted model (Model 3: HR 1.38, 95 % CI 0.86-2.23; P = 0.2; EPV = 4), HR estimates for CH remained directionally consistent but lacked statistical significance (Supplemental Table 1). In models incorporating age, sex and PAD (Model 4: HR 1.57, 95 % CI 1.06-2.32; P = 0.02; EPV = 5.2) or DM (Model 5: HR 1.44, 95 % CI 0.98-2.12; P = 0.06; EPV = 5.2) HRs indicated independent effects of CH on increased risk of MACE at borderline significance (Supplemental Table 1); however, given the low EPV, these results need to be considered exploratory. Furthermore, complete-case deletion was applied, making the analytic samples vary across these models. Of note, we did not observe any significant associations between CH and characteristics of the prevalent carotid artery stenosis as determined by morphological and histological investigations of plaque features (Table 1).

### 3.3 Prognostic significance of mutations in individual CH-associated genes

We next evaluated contributions of the individual genes included in our analysis, excluding patients with mutations detected in more than one gene. Kaplan-Meier analyses for MACE as primary endpoint at 5-year follow-up, comparing patients carrying mutations restricted to either *DNMT3A* (patients available for analysis: n = 6), *TET2* (n = 2) or the group of other mutated genes (n = 5; *ASXL1, CBL, EZH2, KIT* and *ZRSR2*) with patients lacking any detected mutations revealed increased rates of MACE in all mutated subgroups, reaching statistical significance for the group of five (Fig. 4). Furthermore, we addressed a potential impact of clone size (as reflected by VAF) on MACE. For this, we focused on patients who reached the primary endpoint during 5-year follow-up. Analyzing VAFs in patient groups stratified by survival time, we did not detect any dependencies of MACE on clone size (Supplemental Figure 5).

**Fig. 4:**
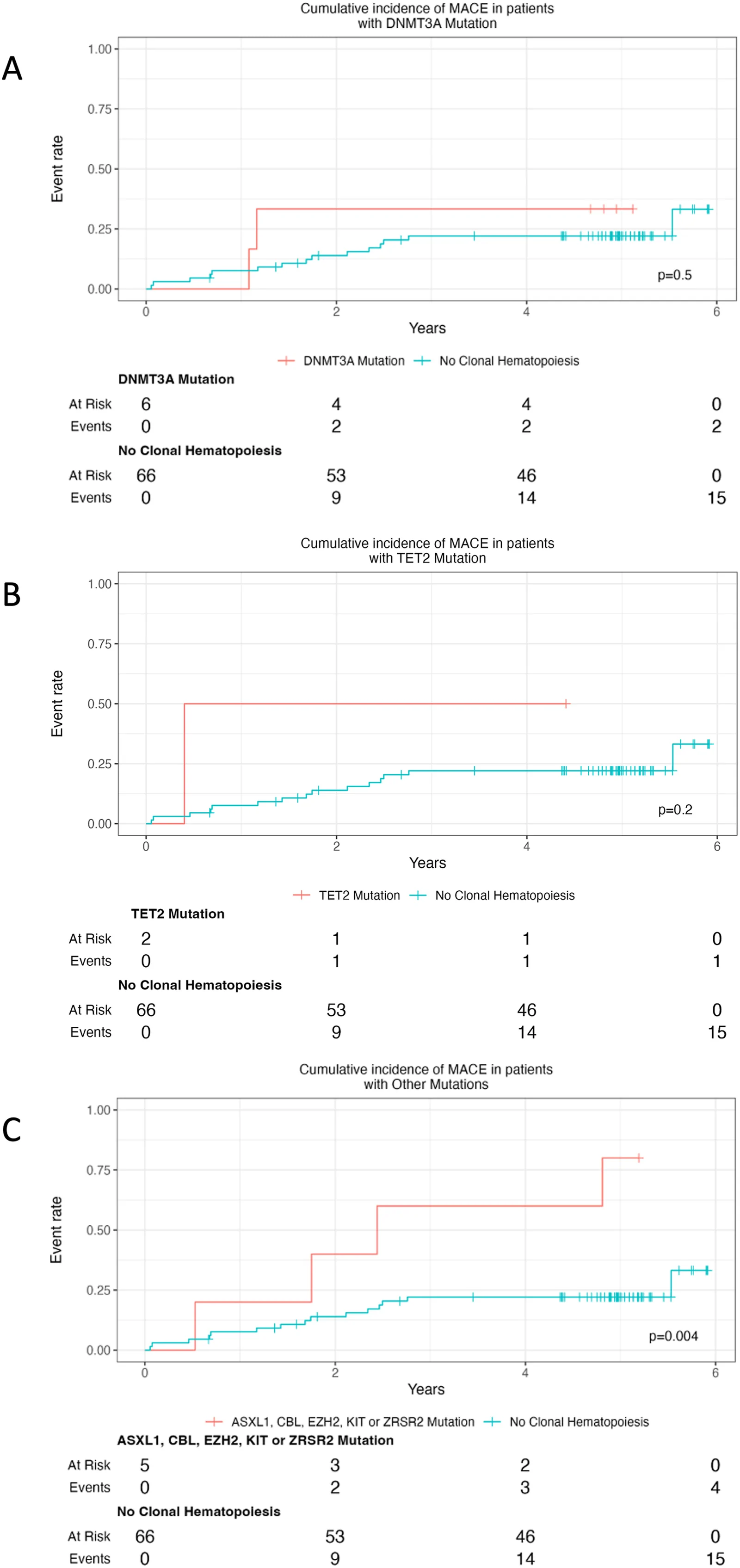
**Mutations in *DNMT3A*, *TET2* and other genes individually affect MACE.** Cumulative incidence of MACE occurrence, comparing patients carrying mutations restricted to either *DNMT3A* (**A**), *TET2* (**B**) or one of the other mutated genes (**C**) with patients lacking any detected mutations.

### 3.4 Differential effects of clonal hematopoiesis based on degree of stenosis

A recent study by Jäger et al. reported combined effects of CH and the degree of carotid stenosis in a patient cohort with asymptomatic, lower-grade stenosis [26]. Building on those findings, we hypothesized that in our cohort of patients with high-grade carotid stenosis, adverse events associated with CH might also correlate with the extent of carotid narrowing or specific features of the atherosclerotic plaque. In our patient cohort, 76 % of patients exhibit at least 80 % stenosis in the diseased carotid artery, precluding an analysis at the NASCET-derived 50 % threshold for hemodynamically significant stenosis. To test for potential correlations between CH and stenosis severity in our high-grade stenosis CEA cohort, we stratified based on a 90 % stenosis threshold (defining patients with highest degree of carotid stenosis), thereby creating balanced, maximally powered groups for sub-cohort analyses. Notably, Kaplan-Meier analyses revealed that the impact of CH on MACE was particularly pronounced in patients with <90 % carotid stenosis, whereas no such effect was observed in those with >90 % stenosis (Supplemental Figure 6). Consistent with these findings, inclusion of an interaction term into a Cox proportional hazards model adjusted for age and sex suggested an effect modification (adjusted interaction HR = 0.39, 95 % CI 0.15 - 1.02, Wald *P* = 0.06; LRT *P* = 0.03), indicating that the association between CH and MACE was weaker in patients with >90 % than in those with <90 %. However, the wide confidence interval indicates limited precision, and the low EPV (4) suggests a substantial risk of model instability.

## 4 Discussion

CH is typically detected by deep sequencing of peripheral blood DNA. Prior studies have used low-coverage whole-exome sequencing or in-house developed CHIP gene panels. In contrast, this study employs a diagnostic-grade 30-gene NGS assay to determine the prevalence and prognostic relevance of CH in patients with high-grade carotid artery stenosis. In our cohort of 83 patients, 20 somatic mutations were identified in 16 individuals (19.7 %), with DNMT3A and TET2 representing the most frequently mutated genes. In line with previous reports [27,28], the majority of patients carried only a single mutated clone.

Addressing a critical evidence gap regarding hematologic determinants of residual cardiovascular risk, our study found that presence of CH was associated with a significantly higher incidence of MACE in patients with both symptomatic and asymptomatic high-grade carotid artery stenosis, followed over a 5-year period. This is in line with previously reported adverse effects of CH on prognosis in patients with ST-segment elevation myocardial infarction (STEMI), aortic stenosis, HF, cardiogenic shock, ischemic stroke as well as in the broader population of individuals with established ASCVD [10,11,26,[29], [30], [31], [32]]. In a cohort of STEMI patients, the presence of DNMT3A- or TET2-mutated CH was associated with worse clinical outcomes but did not affect the extent of CAD, as measured by the rates of 1-, 2-, or 3-vessel involvement [29]. In patients undergoing coronary angiography, CH was associated with an increased risk of all-cause mortality and heart failure; however, findings regarding its correlation with the angiographic burden of CAD have been inconsistent across studies [33,34]. The increased risk of MACE following CEA observed in this study may be mediated by inflammatory mechanisms. Consistent with recent reports [16,35], these effects could reflect the intricate interactions between modulated plaque smooth muscle cells and infiltrating macrophages originating from patients’ PB.

While significant differences in size and sensitivity across NGS-based assays complicate direct comparisons between studies of CH, the frequency of CH observed in our cohort of patients with high-grade carotid stenosis is notably higher than that found in most population-based and cardiovascular cohorts. In patients with PAD from the UK Biobank and Mass General Brigham Biobank, the prevalence of CH carriers was reported as 5.8 % and 5.4 %, respectively, based on whole-exome sequencing [36]. Similarly, a prevalence of 5.1 % was observed in patients with established ASCVD in the UK Biobank [10]. In a notably higher range, beyond patients with STEMI 12.4 % were found to carry CH-driver mutations in DNMT3A or TET2 [29]. Similarly, a recent study described a CH prevalence of 13.7 %, driven by mutations in DNMT3A, TET2, ASXL1, and JAK2, among individuals with asymptomatic carotid atherosclerosis [26]. Investigating a total of 30 genes in patients with high-grade stenosis, we detected CH in 19.7 % of patients at a median age of 71 years, allowing for comparison with the Bioimage cohort (median age 70 years) analyzed by Jaiswal et al. [9], where CH was reported in 11.9 %. Thus, the fraction of CH carriers in our cohort tends to be higher compared to other ASCVD cohorts, but aligns with rates reported in high-risk cardiovascular cohorts, including patients undergoing cardiac procedures involving catheterization (18 % CH) [34] or acute brain ischemia (21 % CH) [13]. Overall, the ∼20 % prevalence of CH in our cohort may reflect an advanced ASCVD state in conjunction with a greater analytical performance of the diagnostic-grade assay compared with other methodologies. Of note, an increasing amount of factors need to be considered when comparing CH prevalence over different cohorts, as recent studies included VAFs below 2 % to call CH in patients with ischemic HF [11], stroke [13,30], and dilated cardiomyopathy [37].

The 30-gene-assay applied on our study revealed the highest number of somatic mutations in the *DNMT3A* gene, followed by *TET2* (Fig. 1B), reflecting previous observations in other cohorts [9,26,27]. Both genes act as critical epigenetic regulators of transcription while other genes driving CH in our cohort (n = 5; i.e. *ASXL1, CBL, EZH2, KIT* and *ZRSR2*) represent a functionally more heterogeneous group, sharing their role in regulation of hematopoiesis [8,38]. When analyzing the impact of mutations within individual genes on MACE, formal statistical significance was restricted to the group of five non-recurrent driver genes; however, there is a trend for increased cardiovascular mortality also in carriers of mutations in *DNMT3A* and *TET2* (Fig. 4). This is in line with recently published data based on a 4-gene-assay (i.e. *DNMT3A, TET2, ASLX1* and *JAK2*) where the effect of CH was reflected by all four individual genes at differential statistical significance [26].

Larger CH clones have previously been more strongly associated with adverse clinical outcomes [27,36,39], with a VAF threshold of 10 % commonly used to define larger clones. In our cohort, clone size and the occurrence of MACE were not linked (Supplemental Figure 5). This might be due to statistical limitations based on limited number of patents that reached the primary endpoint. Alternatively, this may be attributable to the fact that the majority of mutations in our cohort exhibited VAFs between 2 % and 10 %.

In an exploratory pilot analysis stratifying patients according to degree of carotid stenosis at highest grades, we observed a potential association between CH and increased MACE rates predominantly in patients with <90 % stenosis, whereas this pattern was not apparent in those with more severe stenosis (Supplemental Figure 6). Notably, MACE primarily occurred among patients with lower degrees of stenosis referred for CEA, and within this group, CH carriers were disproportionately affected. An interaction model suggested possible effect modification between CH and stenosis group. Although the interaction term did not reach formal statistical significance by Wald testing, results from an LRT were significant. Given the limited precision, these findings should be considered exploratory and require confirmation in larger cohorts to establish whether this observation reflects a true clinical phenomenon or is attributable to sample size limitations.

The relatively small sample size of 83 patients clearly constitutes a limitation of our study. We aimed for mitigating this by applying a comprehensive diagnostic-grade NGS panel, which enabled the accurate screening of 30 genes rather than only a few selected genes commonly used in similar studies [26,40]. The statistically significant associations identified in our study highlight the relevance of CH-associated mutations detected across seven different genes for predicting clinical outcomes. Moreover, the observed effects, even within this relatively small cohort, underscore the role of CH as a strong prognostic factor in ASCVD.

## 5 Conclusions

In conclusion, the presence of CH was associated with an adverse 5-year outcome in this cohort, evidenced by an increased rate of MACE in patients with high-grade carotid artery stenosis undergoing CEA. These results highlight the contribution of CH to residual cardiovascular risk and indicate that commercially available diagnostic-grade NGS panels, routinely used in the evaluation of myeloid neoplasms, can be applied in patients with ASCVD to detect CH and support comprehensive risk stratification. Further studies with larger sample sizes will be required to confirm these findings.

## Consent to participate

All participants provided written consent to participate in the study.

## Ethical considerations

The study conforms to the principles indicated in the Declaration of Helsinki and follows the principles of Good Clinical Practice. The study was approved by the Ethics Committee of the Medical University of Vienna (Ethikkommission der Medizinischen Universität Wien: ethics votum number 2449/2020).

## Consent for publication

Not applicable.

## Data availability

The datasets used and/or analyzed during the current study are available from the corresponding authors on reasonable request.

## Clinical trial number

Not applicable.

## Author contributions

O.H., R.J., H.E., S.S. and S.D. designed the research; O.H., R.J., M.G., H.E. and S.D. conducted experiments and acquired data; O.H., R.J., S.S, and S.D. performed research and data analysis; S.K., M.C.N., J.W., C.H., B.H., C.N. and S.S. provided patient samples or clinical data; O.H., R.J. and S.D. wrote the manuscript; all authors reviewed the manuscript and approved its submission. The order of listing the shared first authors (O.H. and R.J.) was determined alphabetically.

## Funding statement

This project was supported by a research grant of the Austrian Heart Foundation (Österreichischer Herzfonds) awarded to Roland Jäger.

## Declaration of conflicting interest

The authors state no conflict of interest.

## List of abbreviations

ACE: angiotensin-converting-enzyme
ASCVD: atherosclerotic cardiovascular disease
AMI: acute myocardial infarction
AHA: American Heart Association
CAD: coronary artery disease
CH: clonal hematopoiesis
CI: confidence interval
CHIP: clonal hematopoiesis of indeterminate potential
CVD: cardiovascular disease
CEA: carotid endarterectomy
COPD: chronic obstructive pulmonary disease
CRP: C-reactive protein
BMI: body mass index
DM: diabetes mellitus
EPV: events-per-variable
HDL: high-density lipoprotein
hsCRP: high-sensitive C-reactive protein
LDL: low-density lipoprotein
LRT: likelihood-ratio test
MACE: major adverse cardiovascular events
MPN: myeloproliferative neoplasms
NGS: next-generation sequencing
NOAC: new oral anticoagulants
PAD: peripheral artery disease
STEMI: ST-segment elevation myocardial infarction
TIA: transient ischemic attack
VAF: variant allele frequency
