# Supplementary material for: Clonal hematopoiesis is associated with adverse events in patients with high-grade carotid artery stenosis
Source: Atheroscler Plus. 2026 Oct 1;66:100617. doi: 10.1016/j.athplu.2026.100617 (PMC13677976; doi:10.1016/j.athplu.2026.100617)
Supplement: Supplementary file 1 [file mmc1.docx]

Supplemental Material

**Clonal Hematopoiesis is Associated with Adverse Events in Patients with High-Grade Carotid Artery Stenosis**

Olesya Harkot^1,*^, Roland Jäger^2,*,#^, Stephanie Kampf^3^, Michael Gurbisz^2^, Maja Carina Nackenhorst^4^, Johann Wojta^1,5,6^ Christian Hengstenberg^1^, Bernhard Haring^7^, Harald Esterbauer^2^, Christoph Neumayer^3^, Stefan Stojkovic^1^, Svitlana Demyanets^2,8,#^

^1^Division of Cardiology, Department of Internal Medicine II, Medical University of Vienna, Vienna, Austria

^2^Department of Laboratory Medicine, Medical University of Vienna, Vienna, Austria.

^3^Division of Vascular Surgery, Department of Surgery, Medical University of Vienna, Vienna, Austria

^4^Department of Pathology, Medical University of Vienna, Vienna, Austria.

^5^Ludwig Boltzmann Institute for Cardiovascular Research, Vienna, Austria

^6^Core Facilities, Medical University of Vienna, Vienna, Austria

^7^Department of Medicine IV, Clinic Hietzing, Vienna Healthcare Group, Vienna, Austria

^8^Department of Laboratory Medicine, Clinic Hietzing, Vienna Healthcare Group, Vienna, Austria.

*These authors share first authorship.

^#^These authors share correspondence.

# **Supplemental Figures**

**Supplemental Figure 1:** Characteristics of somatic mutations identified in 16 out of 83 patients with high-grade stenosis. **(A)** Distribution of mutations (n=20) within patients with CH (n=16). Blue bars represent the percentage of patients with CH carrying one, two or three mutations. Numbers at bars represent the total count of individuals in groups. **(B)** Frequency plots showing variant allele frequencies (VAFs) for identified mutations (n=20) in different genes (DNMT3A, TET2, others (ASXL1, CBL, EZH2, KIT, SF3B1, TP53, and ZRSR2)). Numbers above bars represent the total number of mutations contained in bins.

**Supplemental Figure 2:** Cox Proportional Hazard Regression Analyses to Assess the Association between MACE and Clonal Haematopoiesis: Univariable Model. Hazard ratios (HRs) per 1-SD for MACE in conjunction with 95% confidence intervals (CI).

**Supplemental Figure 3:** Cox Proportional Hazard Regression Analyses to Assess the Association between MACE and Clonal Haematopoiesis: Multivariable Model corrected for age and sex. Hazard ratios (HRs) per 1-SD for MACE in conjunction with 95% confidence intervals (CI).

**Supplemental Figure 4:** Cox Proportional Hazard Regression Analyses to Assess the Association between MACE and Clonal Haematopoiesis: Multivariable Model corrected for age, sex, peripheral artery disease and active smoking status. Hazard ratios (HRs) per 1-SD for MACE in conjunction with 95% confidence intervals (CI).

**Supplemental Figure 5:** Relationship between clone size and time to MACE as primary endpoint. CH carriers with MACE during follow up (n=7) were stratified into two groups, based on the median survival. Variant allele frequencies (VAFs) for both groups are plotted. Groups were compared using the Mann-Whitney-U test. n.s.: non-significant.

**Supplemental Figure 6:** Effects of CH on cumulative incidence of MACE in patients based on degree of carotid artery stenosis. Incidence of MACE in patients with <90% (A) and ≥90% (B) carotid narrowing.

# **Supplemental Table**

**Supplemental Table 1:** Association of MACE with Clonal Hematopoiesis

| **Model** | **HR (95% CI)** | **N analyzed** | **Events** | **Number of estimated parameters** | **EPV** |
| --- | --- | --- | --- | --- | --- |
| Univariable Model | 1.43 (1.00-2.04) | 81 | 22 | 1 | 22.0 |
| Multivariable Model 1 ^a^ | 1.57 (1.08-2.27) | 81 | 22 | 3 | 7.3 |
| Multivariable Model 2 ^b^ | 1.33 (0.81-2.20) | 55 | 15 | 6 | 2.5 |
| Multivariable model 3 ^c^ | 1.38 (0.86-2.23) | 56 | 16 | 4 | 4.0 |
| Multivariable model 4 ^d^ | 1.57 (1.06-2.32) | 80 | 21 | 4 | 5.3 |
| Multivariable model 5 ^e^ | 1.44 (0.98-2.12) | 79 | 21 | 4 | 5.3 |
| Interaction (unadjusted) ^f^ | 0.39 (0.15-1.01) | 76 | 20 | 3 | 6.7 |
| Interaction (adjusted) ^g^ | 0.39 (0.15-1.02) | 76 | 20 | 5 | 4.0 |

a) Multivariable model 1 adjusted for age and sex. The number of patients with missing data for each covariate is indicated in parentheses: age (0), sex (0). Hazard ratios (HRs) per 1-SD with 95% confidence intervals (CI).
b) Multivariable model 2 adjusted for age, sex, active smoking status, peripheral arterial disease, and diabetes mellitus. The number of patients with missing data for each covariate is indicated in parentheses: age (0), sex (0), active smoking status (25), peripheral arterial disease (1), diabetes mellitus (2). Hazard ratios (HRs) per 1-SD with 95% confidence intervals (CI).
c) Multivariable model 3 adjusted for age, sex, and active smoking status. The number of patients with missing data for each covariate is indicated in parentheses: age (0), sex (0), active smoking status (25). Hazard ratios (HRs) per 1-SD with 95% confidence intervals (CI).
d) Multivariable model 4 adjusted for age, sex, and peripheral arterial disease. The number of patients with missing data for each covariate is indicated in parentheses: age (0), sex (0), peripheral arterial disease (1). Hazard ratios (HRs) per 1-SD with 95% confidence intervals (CI).
e) Multivariable model 5 adjusted for age, sex, and diabetes mellitus. The number of patients with missing data for each covariate is indicated in parentheses: age (0), sex (0), diabetes mellitus (2). Hazard ratios (HRs) per 1-SD with 95% confidence intervals (CI).
f) Unadjusted interaction model including CH carrier status, stenosis grade (categorical, ≥90% vs. <90%) and their interaction term (i.e. the differential effect of CH across stenosis strata). The number of patients with missing data for each covariate is indicated in parentheses: CH carrier status (0), stenosis grade (5). Hazard ratios (HRs) per 1-SD with 95% confidence intervals (CI).
g) Interaction model as in (f), additionally adjusted for age and sex. The number of patients with missing data for each covariate is indicated in parentheses: CH carrier status (0), stenosis grade (5), age (0), sex (0). Hazard ratios (HRs) per 1-SD with 95% confidence intervals (CI).

No corrections for multiple testing were applied. EPV: Events per variable.
